# Supplementary material for: Biochemical and Hematological Correlates of Elevated Homocysteine in National Surveys and a Longitudinal Study of Urban Adults
Source: Nutrients. 2020 Mar 30;12(4):950. doi: 10.3390/nu12040950 (PMC7230768; doi:10.3390/nu12040950)
Supplement: Supplementary file 1 [file nutrients-12-00950-s001.pdf]

ON-LINE SUPPLEMENTARY MATERIAL

Biochemical and hematological correlates of elevated homocysteine in national surveys and a longitudinal study of urban adults

Beydoun et. al.

Supplementary Figure 1. Participant flowchart

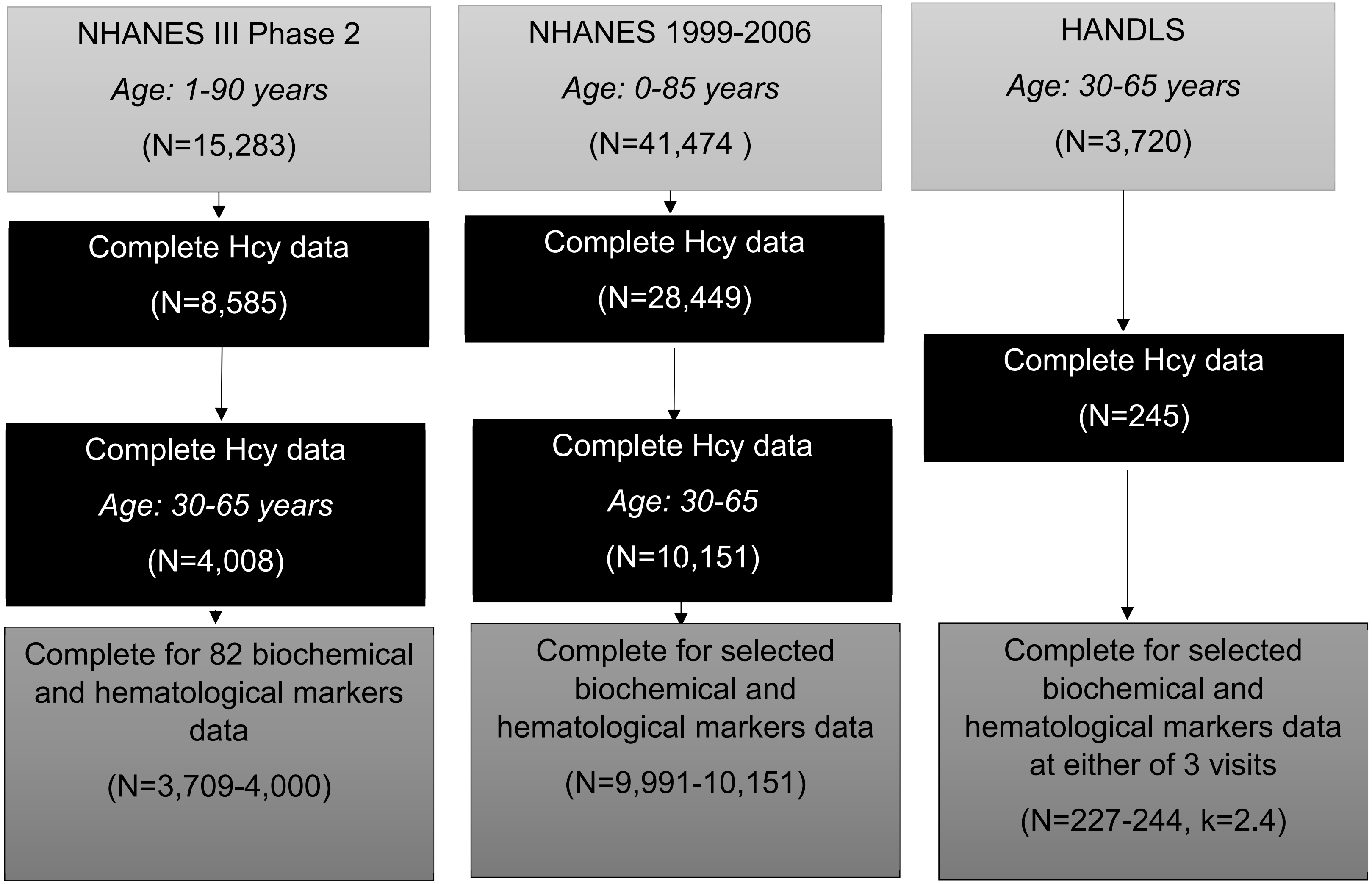

Abbreviations: k=Average number of repeats/participant; HANDLS=Healthy Aging in Neighborhoods of Diversity Across the Life Span; Hcy=Homocysteine; N=number of participants; NHANES=National Health and Nutrition Examination Surveys.

SUPPLEMENTAL METHODS 1: BIOCHEMICAL AND HEMATOLOGICAL INDICES: NHANES III, phase 2: 1991-1994

BIOMARKER	DESCRIPTION (UNITS)
hopsi	Serum homocysteine: SI (umol/L)
cop	Serum cotinine (ng/mL)
vdpsi	Serum vitamin D: SI (nmol/L)
t4psi	Serum throxine: SI (nmol/L)
thpsi	Serum thyroid stim hormone: SI (mU/L)
tmp	Serum antimicrosomal antibody (U/mL)
tap	Serum anti-thyroglobulin antibody (U/mL)
wcpsi	White blood cell count: SI
lmppcnt	Lymphocyte percent (Coulter)
moppcnt	Mononuclear percent (Coulter)
grppcnt	Granulocyte percent (Coulter)
lmp	Lymphocyte number (Coulter)
mop	Mononuclear number (Coulter)
grp	Granulocyte number (Coulter)
rcpsi	Red blood cell count: SI
hgpsi	Hemoglobin: SI (g/L)
htpsi	Hematocrit: SI (L/L = 1)
mvpsi	Mean cell volume: SI (fL)
mcpsi	Mean cell hemoglobin: SI (pg)
mhpsi	Mean cell hemoglobin concentration: SI
rwpsi	Red cell distribution width: SI (fraction)
plpsi	Platelet count: SI
dwp	Platelet distribution width (%)
pvpsi	Mean platelet volume: SI (fL)
pbpsi	Lead: SI (umol/L)
eppsi	Erythrocyte protoporphyrin: SI (umol/L)
fepsi	Serum iron: SI (umol/L)
tipsi	Serum TIBC: SI (umol/L)
pxp	Serum transferrin saturation (%)
frpsi	Serum ferritin: SI (ug/L)
fopsi	Serum folate: SI (nmol/L)
rbpsi	RBC folate: SI (nmol/L)
vbpsi	Serum vitamin B12: SI (pmol/L)
vcpsi	Serum vitamin C: SI (mmol/L)
icpsi	Serum normalized calcium: SI (mmol/L)
capsi	Serum total calcium: SI (mmol/L)

sepsi	Serum selenium: SI (nmol/L)
vapsi	Serum vitamin A: SI (umol/L)
vepsi	Serum vitamin E: SI (umol/L)
acpsi	Serum alpha carotene: SI (umol/L)
bcpsi	Serum beta carotene: SI (umol/L)
bxpsi	Serum beta cryptoxanthin: SI (umol/L)
lupsi	Serum lutein/zeaxanthin: SI (umol/L)
lypsi	Serum lycopene: SI (umol/L)
repsi	Serum sum retinyl esters: SI (umol/L)
tcpsi	Serum cholesterol: SI (mmol/L)
tgpsi	Serum triglycerides: SI (mmol/L)
hdpsi	Serum HDL cholesterol: SI (mmol/L)
crp	Serum C-reactive protein (mg/dL)
ahp	Serum hepatitis A antibody
hbp	Serum hepatitis B core antibody
hcp	Serum hepatits C antibody
rupunit	Serum rubells antibody (IU)
napsi	Serum sodium: SI (mmol/L)
skpsi	Serum potassium: SI (mmol/L)
clpsi	Serum chloride: SI (mmol/L)
c3psi	Serum bicarbonate: SI (mmol/L)
scpsi	Serum total calbicarcium: SI (mmol/L)
pspsi	Serum phosphorus: SI (mmol/L)
uapsi	Serum uric acid: SI (umol/L)
sgpsi	Serum glucose: SI (mmol/L)
bupsi	Serum blood urea nitrogen: SI (mmol/L)
tbpsi	Serum total bilirubin: SI (umol/L)
cepsi	Serum creatinine: SI (umol/L)
sfpsi	Serum iron: SI (umol/L)
chpsi	Serum cholesterol: SI (mmol/L)
trpsi	Serum triglycerides: SI (mmol/L)
aspsi	Aspartate aminotransferase: SI (U/L)
atpsi	Alanine aminotransferase: SI (U/L)
ggpsi	Gama glutamyl transferase: SI (U/L)
ldpsi	Serum lactate dehydrogenase: SI (U/L)
appsi	Serum alkaline phosphatase: SI (U/L)
tppsi	Serum total protein: SI (g/L)
ampsi	Serum albumin: SI (g/L)
gbpsi	Serum globulin: SI (g/L)
ospsi	Serum osmolality: SI (mmol/Kg)
ghp	Glycated hemoglobin: (%)

g1p	Plasma glucose (mg/dL)
g1psi	Plasma glucose: SI (mmol/L)
udpsi	Urinary cadmium: SI (nmol/L)
urpsi	Urinary creatinine: SI (mmol/L)
ubp	Urinary albumin (ug/mL)
uip	Urinary iodine (ug/dL)

Homocysteine

Homocysteine concentrations were measured as part of an NHANES III surplus sera project on serum samples from participants 12 years of age or older who were seen during phase II of this survey (1991–1994). This project is described in greater detail elsewhere[1]. Homocysteine concentrations were measured at the Jean Mayer USDA Human Nutrition Research Center on Aging at Tufts University by using the high-performance liquid chromatography method of Araki and Sako[2]. The interassay coefficient of variation for this assay was 6%.

Hematological indices

Red blood, White blood and platelet cell indices

Microscopic examination (manual differential) of the peripheral blood spread on a glass slide utilized a stained blood film to perform a differential leukocyte count, evaluate red cell morphology, and estimate number of platelets. Manual differential variables include segmented neutrophils, lymphocytes, monocytes, eosinophils, basophils, blasts, promyelocytes, metamyelocytes, myelocytes, bands, atypical lymphocytes, anisocytosis, basophilic stippling, hypochromia, poikilocytosis, polychromatophilia, macrocytosis, microcytosis, sickle cells, spherocytosis, target cells, toxic granulation, and vacuolated cells (GRPDIF, LMPDIF, MOPDIF, EOP, BAP, BOP, BLP, PRP, MEP, MLP, BAP, LAP, ANP, BSP, HZP, PKP, POP, MRP, MIP, SIP, SHP, TTP, TXP, and VUP).

In NHANES III, a manual differential was performed on a special subsample of examinees aged one year and older. This manual differential was used for internal quality control purposes and to confirm abnormal hematology results. This subsample was defined as a random 10-percent sample of all examined persons plus all examinees who had a predetermined high or low value for one or more of the following hematologic assessments: white blood cell count (WBC), red blood cell count (RBC), hemoglobin, hematocrit, mean cell volume (MCV), mean cell hemoglobin (MCH), mean cell hemoglobin concentration (MCHC), red blood cell distribution width (RDW), platelet count, mean platelet volume (MPV), lymphocyte percentage, mononuclear percentage, or granulocyte percentage. A table of predetermined high and low values for WBC, RBC, hemoglobin, hematocrit, MCV, MCH, MCHC, RDW, platelet count, MPV, lymphocyte percentage, mononuclear percentage, and granulocyte percentage is located in the Manual for Medical Technicians[3].

RCP: Red blood cell count
See notes for HGP and GRP.

VUP: Vacuolated cells

See note for ANP.

WCP: White blood cell count
See note for HGP and GRP.

(A) Biochemical indices

Blood micronutrient and electrolyte biomarkers

Serum 25-hydroxyvitamin D (25(OH)D)

The INCSTAR 25(OH)D assay consists of a two-step procedure, with the first being a rapid extraction of 25(OH)D and other hydroxylated metabolites from serum or plasma with acetonitrile, and the second being the assay of the treated sample by using an equilibrium RIA procedure. [4]

Serum ferritin

Ferritin, like hemoglobin, is a major iron storage protein. Serum ferritin levels increase as a result of iron overload, aging, infection, inflammation, liver disease, juvenile rheumatoid arthritis, leukemia, and Hodgkin's disease; and decrease as a result of iron deficiency. Ferritin is measured by using the Bio-Rad Laboratories' "QuantImune Ferritin IRMA" kit, a single-incubation two-site immunoradiometric assay (IRMA) based on the general principles of assays. [4,5]

Serum folate and vitamin C

Folate is required in cellular metabolism and hematopoiesis, and prolonged deficiency leads to megaloblastic anemia. In NHANES III, serum folate is measured by using the Bio-Rad Laboratories "Quantaphase Folate" radioassay kit.[4,6]

Serum normalized calcium

To measure the total calcium concentration, protein-bound and complexed calcium must be released.[4] Within the pH range of 6.9-8.0, the NOVA 7 analyzer can predict the normalized calcium level within 2% of the theoretical value.[7]

Serum selenium

Serum selenium is measured by atomic absorption spectrometry[4] in a procedure based on Lewis et al.[8] and Paschal and Kimberly.[9]

Serum vitamin E, retinol, retinyl esters, carotenoids

Serum levels of vitamin A (retinol), vitamin E (α-tocopherol), retinyl esters and carotenoids are measured by isocratic high performance liquid chromatography with detection at three wavelengths, namely 300, 325, and 450 nm. Quantitation is accomplished by comparison of peak heights with a standard solution.[4]

LUP: Serum lutein/zeaxanthin

The lower limit of detection (LOD) for lutein/zeaxanthin was 0.43 ug/dL. Using the LOD coding formula (detection limit divided by the square root of two), the calculated value indicating that the serum lycopene results were below the level of detection would be 0.30. After rounding, the value of 0 (zero) was placed in the results field to indicate that the serum lutein/zeaxanthin was below 0.43 ug/dL.

LYP: Serum lycopene

The lower limit of detection (LOD) for lycopene was 0.63 ug/dL. Using the LOD coding formula (detection limit divided by the square root of two), the calculated value indicating that the serum lycopene results were below the level of detection would be 0.44. After rounding, the value of 0 (zero) was placed in the results field to indicate that the serum lycopene was below 0.63 ug/dL.

FEP: Serum iron

Laboratory methods differed between NHANES III and previous surveys. Therefore, results may not be comparable between surveys. Consult the Laboratory Procedures Used for NHANES III (U.S. DHHS, 1996).

PXP: Serum transferrin saturation

This value was calculated as (FEP/ TIP) * 100.

RBP: RBC folate

See note for FOP.

SEP: Serum selenium

Selenium values were measured on two Perkin-Elmer graphite furnace atomic absorption spectrophotometers (model 3030 and model 5100) during the six-year study. Based on a comparability study using linear models, the results generated using the Model 5100 instrument (from 12/07/90 to 1/13/95) were on average 4.3 percent higher than those from the Model 3030 instrument (used from 10/1/88 to 12/06/90).Since the Model 5100 represented more precise measurements, the model 3030 data were adjusted to make them comparable to the Model 5100. Perkin-Elmer Model 5100 Zeeman-corrected graphite furnace atomic absorption spectrophotometer testing began on 12/07/90. All selenium values measured prior to 12/07/90 were adjusted to the AA5100 values. The formula used was:

New value = 16.795 + 0.902 * original value.

SFP: Serum iron

This value was obtained from the standard battery of biochemical assessments. Use of the laboratory test result from the reference method (FEP), rather than the SFP value, is generally recommended. For most analyses of serum iron, the appropriate variable to use will be FEP. The value from the biochemistry profile (SFP) should not be used routinely. Consult the Laboratory Procedures Used for NHANES III

(U.S. DHHS, 1996) for details. Laboratory test results for SFP were added to the protocol after NHANES III began. This result field was blank-filled for examinees who were examined prior to the start of testing.

VCP: Serum vitamin C

For NHANES III, serum concentrations of vitamin C were measured using a total vitamin C, fully reduced method using high-performance liquid chromatography with electrochemical detection (HPLC-EC) analysis.

VEP: Serum vitamin E
The vitamin E value of 9999 was confirmed.

Blood metabolic and inflammatory indices

Thyroid hormones

Total (protein-bound and free) circulating thyroxine (T_4) concentrations were determined using an enzyme-based homogeneous immunoassay on the Hitachi 704. The reference range is 4.5 to 12.5 μg/dL. The coefficient for inter-assay coefficient of variation (CV) was less than 10%. [4]

The thyroid stimulating hormone (TSH, in mu/L) assay is a chemiluminescence immunometric assay utilizing a mouse monoclonal antibody to TSH immobilized on a polystyrene bead and a goat polyclonal antibody to TSH conjugated with an acridinium ester. The reference range is 0.3 to 5.0 mu/L. The coefficient for inter-assay CV was less than 5%. [4]

Serum lipids: total cholesterol (TC), triglycerides (TG) and high-density lipoprotein-Cholesterol (HDL-C)

Cholesterol is measured enzymatically in serum or plasma in a series of coupled reactions that hydrolyze cholesterol esters and oxidize the 3-OH group of cholesterol. Triglycerides are measured using a series of coupled reactions in which triglycerides are hydrolyzed to produce glycerol. HDL-cholesterol is measured following the precipitation of the other lipoproteins with a polyanion/divalent cation mixture. [4]

CHP: Serum cholesterol
This value was obtained from the standard battery of biochemical assessments. Use of the laboratory test result from the reference method (TCP), rather than the CHP value, is generally recommended.For most analyses of serum cholesterol, the appropriate variable to use will be TCP. The value from the biochemistry profile (CHP) should not be used routinely. Consult the Laboratory Procedures Used for NHANES III (U.S. DHHS, 1996) for the details.

LCP: Serum LDL cholesterol calculation
The value for LDL was calculated by the Friedewald equation as follows:
LDL = total cholesterol - high density cholesterol - triglyceride/5.
Because the equation is not valid when serum triglyceride values exceed 400 mg/dL, the LDL is missing when serum triglyceride (TGP) exceeds 400 mg/dL.
Serum LDL was calculated on examinees who were instructed to fast

(ages 12 and older) and who did fast at least nine hours, were examined in the morning, and were randomly assigned to the morning fasting sample (WTPFHSD6 > 0). Therefore, LDL would be blank if examinees were aged less than 12 years, fasted fewer than nine hours, were examined in an afternoon or evening session, or were not randomly assigned to the morning session. For the purpose of this calculation, the number of hours fasted was rounded to the nearest whole integer.
For more information regarding this equation, refer to the Laboratory Procedures Used for NHANES III (U.S. DHHS, 1996).

TGP: Serum triglycerides
Serum triglyceride levels were measured regardless of the examinee's fasting status. Mean serum triglycerides and the distribution of serum triglycerides should be estimated only on examinees who did fast at least nine hours, were examined in the morning, and were randomly assigned to the morning fasting sample (WTPFHSD6 > 0). For the purpose of this calculation, the number of hours fasted was rounded to the nearest whole integer. Consult the Laboratory Procedures Used for NHANES III (U.S. DHHS, 1996) for details.

TRP: Serum triglycerides
This value was obtained from the standard battery of biochemical assessments. Use of the laboratory test result from the reference method (TGP), rather than the TRP value, is generally recommended. For most analyses, the appropriate variable to use is TGP. The value from the biochemistry profile (TRP) should not be used routinely. Consult the Laboratory Procedures Used for NHANES III (U.S. DHHS, 1996) for details. Results for TRP were added to the protocol after NHANES III began. This result field was blank-filled for examinees who were examined prior to the start of testing.

Glucose biomarkers

Serum C-peptide (first venipuncture)

The specimen for this assay was obtained at the time of the initial venipuncture. This result is available for all six years of the survey. Examinees aged 40-74 years who used insulin were excluded from the OGTT. A first venipuncture was obtained, but the glucose challenge and second venipuncture were canceled. In these instances, the variables G1P, C1P and I1P have a value, but the results G2P, C2P and I2P from the second venipuncture are blank-filled to indicate a medical exclusion.

C2P: Serum C-peptide (second venipuncture)

Post-glucose challenge levels of C-peptide and insulin for examinees who had an OGTT were measured only during 1991-1994.

G1P: Plasma glucose (first venipuncture)

Plasma glucose was measured using the reference method on examinees aged 20 years and older. Consult the Laboratory Procedures Used for NHANES III (U.S. DHHS, 1996) for details. During NHANES III, OGTT testing was conducted on MEC examinees aged
40-74 years. A random assignment was made prior to conducting the OGGT to determine who should receive a morning examination[3,10]. As a result, approximately half of the OGGT examinees received the morning OGTT after an overnight fast. This subsample most closely conformed to the World Health Organization (WHO) criteria for OGTT testing to identify diabetes[11]. Therefore, this morning subsample is the NHANES III subsample that should be used to estimate the prevalence of diabetes and impaired glucose tolerance. People who reported a medical history of diabetes but who were not using insulin therapy were asked to conform to the fasting instructions for their examination session and were eligible for an OGTT if the age criteria were satisfied. The morning sample weights (WTPFHSD6) for total NHANES III weights for the morning OGTT subsample should be used when weighting these data to generate national estimates. Data from the afternoon and evening OGTTs do not conform to the WHO protocol for diagnosing diabetes or IGT and should not be used for these purposes.

If an examinee was given an OGTT during an examination session other than the session assigned, that examinee's sample weight for the assigned session will be zero. For example, if an examinee was selected for a morning OGTT but was tested in the afternoon, the examinee's morning sample weight for the OGTT will be zero.

GHP: Glycated hemoglobin (HbA1c)
Glycohemoglobin measurements for NHANES III were performed by the Diabetes Diagnostic Laboratory at the University of Missouri -- Columbia using the Diamat Analyzer System (Bio-Rad Laboratories, Hercules, CA). This ion-exchange HPLC system measures HbA1c (a specific glycohemoglobin) and has demonstrated excellent, long-term precision (interassay CV's 2.0). It was standardized to the reference method that was used for the Diabetes Control and Complications Trial (DCCT). Variant hemoglobins, including hemoglobin C, D, F, and elevated HbF, can interfere with HbA1c measurement by the Diamat HPLC. Hemoglobin S in its heterozygous state does not interfere with this assay. Although interferences usually can be detected by an abnormal Diamat chromatogram, HbA1c results for these specimens were not considered valid. Therefore, samples containing hemoglobin variants or elevated HbF or samples that produce chromatograms indicating hemoglobin degradation were analyzed by an alternate method that used affinity chromatography to separate glycohemoglobin. Affinity chromatographic methods were not affected by the presence of hemoglobin variants and were less sensitive to hemoglobin degradation due to improper sample handling. The affinity method used also was standardized to the DCCT reference method. Reasons for using the affinity method for an examinee's test included an extra peak on the chromatogram, hemoglobin C, elevated hemoglobin F, or other abnormal hemoglobin.

I1P: Serum insulin (first venipuncture)

This is the adjusted insulin value for examinees. Most of the Insulin values in NHANES III (1988-1991) were adjusted because the manufacturer of the laboratory testing kits changed during that period. An indicator of the kit number is located in the I1P2PFLG field (i.e., 1 = Kit 1, 2 = Kit 2, and 3 = Kit 3). All insulin values from Kit 1 and Kit 2 assays were adjusted linearly to match the Kit 3 numbers. Further information on this adjustment procedure is available in the Laboratory Procedures Used for NHANES III (U.S. DHHS, 1996).

The equations used to adjust the data were:
Kit 3 = 0.787 (Kit 1) + 0.832 Equation 1
Kit 3 = 0.597 (Kit 2) + 1.746 Equation 2

The following steps were used to make the adjustment:

1. Equation 1 was applied to group 1 (Kit 1) data
2. Equation 2 was applied to group 2 (Kit 2) data
3. Group 3 data (Kit 3) were left unchanged.

The time periods for the insulin kits were as follows:
Group Assay Period Assay Method
1 10/88-01/05/90 Kit 1
2 01/06/90-09/06/90 Kit 2
3 11/01/90-end of study Kit 3

I2P: Serum insulin (second venipuncture)

SGP: Serum glucose
This value was obtained from the standard battery of biochemical assessments. Use of the laboratory test result for plasma
glucose from the reference method (G1P), rather than the SGP value, is generally recommended. For most analyses, the appropriate variable to use will be G1P. The value from the biochemistry profile (SGP) should not be used routinely.Consult the Laboratory Procedures Used for NHANES III (U.S. DHHS, 1996) for details.

Protein biomarkers

GBP: Serum globulin
Globulin results were added to the protocol after NHANES III began. This result field was blank-filled for examinees who were examined prior to the start of testing.

HGP: Hemoglobin

In NHANES I, NHANES II, and HHANES, determinations of red and white blood cell counts were made using a semiautomated cell counter Coulter model FN). Determinations of hemoglobin concentration (Hb) were made using a Coulter hemoglobinometer, and determinations of packed cell volume (PCV) were made using the microhematocrit centrifuge method. The hematologic indices MCH, MCHC, and MCV were calculated as follows:

MCH = Hb/RBC
MCHC= Hb/PCV
MCV = PCV/RBC

In NHANES III, these hematologic parameters were determined by using a fully automated Coulter S+JR hematology analyzer. These analyzers measured the mean (red) cell volume (MCV) directly, utilizing a process of continuous integration of pulse heights divided by the pulse number; PCV values were calculated through the multiplication of MCV and RBC.

Although it has been shown that identified errors in the microhematocrit method caused by plasma trapping and red cell dehydration approximately compensate each other,[12] packing errors can occur in macrocytic anemia and can be considerable in sickle cell anemia, spherocytosis, and thalassemias[13]. Therefore, individual values for MCV, PCV ("hematocrit"), and MCHC from NHANES III cannot be compared directly to values from the previous NHANES.

HTP: Hematocrit

See note for HGP.

MCPSI: Mean cell hemoglobin
See note for HGP.
MHP: Mean cell hemoglobin concentration
See note for HGP.

CEP: Serum Creatinine

The Cleveland Clinic Foundation (CCF) laboratory analyzed the serum creatinine specimens using a Roche coupled enzymatic assay (creatininase, creatinase, sarcosine oxidase, kits # 1775677 and 1775766) performed on a Roche P Module instrument. The Roche method calibrators were traceable to an isotope dilution mass spectrometric method for serum creatinine using standard references methods (NIST SRM 967) and confirmed by analysis of CAP LN-24 linearity set based on NIST assigned values. Serum creatinine by the Roche method was then compared to the original NHANES III measurements which used the Jaffe kinetic alkaline picrate method performed on a Roche Hitachi 737 analyzer. There were significant differences in results between these two measurements. The comparison of values revealed the mean (SD) serum creatinine at NHANES, CCF, and their difference were 1.177 (0.315), 0.947 (0.302), and 0.231(0.066) mg/dL, respectively (paired t-test, $p<0.0001$). The Deming regression (adjusting for errors in measurement) for the correction is Standard Creatinine (Y) = 0.960*NHANES Creatinine (X) – 0.184 (r = 0.978).

IgG to infections

RUP: Serum rubella antibody

Rubella antibody data are reported both as an optical density index and in International Units. The index was calculated by subtracting the absorbance of the control well from the absorbance of the antigen well (AG-NS) and dividing the difference by the cut-off value. The

cut-off value was calculated as the mean AG-NS value of duplicate 10 IU standards. The equation used was: O.D. index = (AG-NS)/Cut-off value
An O.D. index greater than or equal to one indicates the presence of antibody.

RUPUNIT: Serum rubella antibody (IU)

Rubella antibody data are reported both as an optical density index and in International Units. International Units were calculated based on a standard curve using a regression analysis of duplicate AG-NS values of 10, 40, & 100 IU standards and their squares. An International Unit value greater than or equal to 10 indicates the presence of antibody.

SAP: Serum hepatitis B surface antigen
See note for HBP.

SSP: Serum hepatitis B surface antibody
See note for HBP.

TOP: Serum toxoplasmosis antibody
The presence and quantity of antibody to Toxoplasma gondii in the test sample were determined by comparing the optical density of the test sample to a standard curve. A standard curve was constructed using optical density readings from positive control sera obtained from a kit; these readings were calibrated to WHO Toxo 60 serum and read as International Units (IU/mL). Those test samples exhibiting titer below 7 IU/mL indicated a non-significant level of antibody according to this technique; thus, they were considered to be negative, indicating no infection. Those test samples with results greater than 6 IU/mL were considered to be positive, indicating infection at some undetermined time.

VRP: Serum varicella antibody
Varicella antibody data were reported as an optical density index. See note RUP for the index calculation. The equation used was:
O.D. index = (AG-NS)/Cut-off value
The cut-off value was 0.1. An O.D. index equal to or greater than one indicates the presence of antibody.

Other inflammatory markers

MEP: Metamyelocyte cells
See note for ANP.
MIP: Microcytosis
See note for ANP.
MLP: Myelocyte cells

See note for ANP.

MOP: Mononuclear number

See note for GRP.

MOPDIF: Monocyte cells

See note for ANP.

MRP: Macrocytosis

See note for ANP.

MVPSI: Mean cell volume

See note for HGP.

PLP: Platelet count

See note for GRP.

POP: Polychromatophilia

See note for ANP.

PRP: Promyelocyte cells

See note for ANP.

Liver enzymes

The kinetic assay analyzer computer uses absorbance measurements to calculate alkaline phosphatase (ALP), alanine aminotransferase (ALT), aspartate aminotransferase (AST), BUN, creatinine, gamma glutamyl transaminase (G-GT), and lactate dehydrogenase (LDH) concentrations.

(a) Alkaline phosphatase (ALP)

The method is linear up to 1000 U/L. When reanalyzing any specimen with a concentration greater than 1000 U/L, prepare a 10-fold (1+9) dilution of the specimen with saline.

The minimum detection limit, based on a linear regression curve of certified material analyzed 20 times, is 2 U/L. Results below the detection limit are reported as <2 U/L.

(b) Alanine aminotransferase (ALT)

The method is linear up to 400 U/L. When reanalyzing any specimen with a concentration greater than 400 U/L, prepare a 10-fold (1+9) dilution of the specimen with saline. The minimum detection limit, based on a linear regression curve of certified material analyzed 20 times, is 1 U/L. Results below the detection limit are reported as <1 U/L.

(c) Aspartate aminotransferase (AST)

The method is linear up to 800 U/L. When reanalyzing any specimen with a concentration greater than 800 U/L, prepare a 10-fold (1+9) dilution of the specimen with saline. The results must then be multiplied by 10 to account for this dilution. The minimum detection limit, based on linear a regression curve of certified material analyzed 20 times, is 1 U/L. Results below the detection limit are reported as <1 U/L.

(d) ASPSI: Aspartate aminotransferase

α-Ketoglutarate reacts with L-aspartate in the presence of AST to form L-glutamate plus oxaloacetate. The indicator reaction uses the oxaloacetate for a kinetic determination of NADH consumption. The International Federation of Clinical Chemistry (IFCC) has now recommended standardized procedures for ALT determination, including 1) optimization of substrate concentrations, 2) the use of Tris buffers, 3)

preincubation of a combined buffer and serum solution to allow side reactions with NADH to occur, 4) substrate start (α -ketoglutarate), and 5) optimal pyridoxal phosphate activation.

(e) Gamma glutamyl transaminase (γ-GT)

The method is linear up to 1200 U/L. When reanalyzing any specimen with a concentration greater than 1200 U/L, prepare a 10-fold (1+9) dilution of the specimen with saline. The minimum detection limit, based on a linear regression curve of certified material analyzed 20 times, is 2 U/L. Results below the detection limit are reported as <2 U/L.

(f) Lactate dehydrogenase (LDH)

The method is linear up to 1000 U/L. When reanalyzing any specimen with a concentration greater than 1000 U/L, prepare a 10-fold (1+9) dilution of the specimen with saline. The minimum detection limit, based on a linear regression curve of certified material analyzed 20 times, is 2 U/L. Results below the detection limit are reported as <2 U/L.

Urinary biomarkers

Urinary iodine (IU) is measured by using the reduction-oxidation reaction between ceric and arsenite catalyzed by iodide. Therefore, UI is proportional to its catalytic activity. Urine samples, controls, and iodate standard are digested with chloric acid, then measured spectrometrically at 420 nm with a Technicon AutoAnalyzer. Calculations are based on an iodine standard curve. The final concentration of UI is expressed in μg/dL and is easily converted to μg UI per gram urinary creatinine.[4,14,15]

Urinary creatinine was analyzed with a Jaffé rate reaction using an ASTRA analyzer.[4] Creatinine is released into the plasma at a relatively constant rate and thus has a constant amount per unit muscle mass. Consequently, creatinine is the best indicator of impaired kidney function.

A solid-phase fluorescent immunoassay (FIA) was used for the measurement of urinary albumin.[16] Increased microalbuminuria is a sign of renal disease and is predictive of nephropathy risk in type 2 diabetes patients. [4,16]

Cadmium analysis is used to identify toxicity. Occupational exposure is the most common cause of elevated cadmium levels.[4] Cadmium is measured in urine by atomic absorption spectrometry by using a modification of the method described by Pruszkowska et al. [17]

URP: Urinary creatinine

Although the laboratory method detection limit for urinary creatinine is 1 mg/dL, all values below 10 mg/dL were considered "statistically suspect" and were coded as "below level of detection".

SUPPLEMENTAL METHODS 2: BIOCHEMICAL AND HEMATOLOGICAL INDICES SELECTED FOR NHANES 1999-2006

Total homocysteine (tHcy)

Total homocysteine (tHcy) in plasma is measured by the "Abbott Homocysteine (HCY) assay",[18] a fully automated fluorescence polarization immunoassay (FPIA) from Abbott Diagnostics performed on the AxSYM® platform.[19] In brief, dithiothreitol (DTT) reduces homocysteine bound to albumin and to other small molecules, homocystine, and mixed disulfides, to free thiol. S-adenosyl-homocysteine (SAH) hydrolase catalyzes conversion of homocysteine to SAH in the presence of added adenosine. In the subsequent steps, the specific monoclonal antibody and the fluoresceinated SAH analog tracer constitute the FPIA detection system.[20] Plasma total homocysteine concentrations are calculated by the Abbott AxSYM® using a machine-stored calibration curve.

An international round robin performed in 1998[21] demonstrated that the Abbott method is fully equivalent to other most frequently used methods in this field (i.e., HPLC-FD, HPLC-ED, GC/MS). Thus, the Abbott Homocysteine (HCY) assay will be used as primary method for the determination of plasma total homocysteine in NHANES 1999+. For NHANES 1999-2001, the Abbott IMx® analyzer was used, starting NHANES 2002, the Abbott AxSYM® analyzer is used. The IMx® and the AxSYM® platforms are both using the same reagent kit, but the AxSYM® is a newer fully-automated analyzer that can measure multiple analytes during one run. Pernet et al. showed that the two platforms agree well. [19]

This method is linear for homocysteine in the range 0.8-50 µmol/L. Samples with results <2 µmol/L or >15 µmol/L are reanalyzed for confirmation before results are released. Samples with total homocysteine concentrations >50 µmol/L are diluted 10-fold with PBS or FPIA buffer and reanalyzed. This method has a total coefficient of variation in the range of 3-6%.

Folate/Vitamin B12 (Serum and whole blood)

Both vitamins are measured by using the Bio-Rad Laboratories "Quantaphase II Folate/vitamin B12" radioassay kit [22,23]. The assay is performed by combining serum or a whole blood hemolysate sample with ^{125}I-folate and ^{57}Co-vitamin B12 in a solution containing dithiothreitol (DTT) and cyanide. The mixture is boiled to inactivate endogenous folate-binding proteins and to convert the various forms of vitamin B12 to cyanocobalamin. The reduced folate and its analogs are stabilized by DTT during the heating. The mixture is cooled and then combined with immobilized affinity-purified porcine intrinsic factor and folate-binding proteins. The addition of these substances adjusts and buffers the pH of the reaction mixture to 9.2. The reaction mixture is then incubated for 1 hour at room temperature.

Creatinine

The LX20 modular chemistry side uses the Jaffe rate method (kinetic alkaline picrate) to determine the concentration of creatinine in serum, plasma, or urine. A precise volume of sample is introduced into a reaction cup containing an alkaline picrate solution. Absorbance readings are taken at both 520 nm and

560 nm. Creatinine from the sample combines with the reagent to produce a red color complex. The observed rate measurement at 25.6 seconds after sample introduction has been shown to be a direct measure of the concentration of the creatinine in the sample.

Uric Acid

The LX20 uses a timed endpoint method to measure the concentration of uric acid in serum, plasma, or urine. Uric acid is oxidized by uricase to produce allatoin and hydrogen peroxide. The hydrogen peroxide reacts with 4-aminoantipyrine (4-AAP) and 3.5-dichloro-2-hydroxybenzene sulfonate (DCHBS) in a reaction catalyzed by peroxidase to produce a colored product. The system monitors the change in absorbance at 520 nm at a fixed time interval. The change in absorbance is directly proportional to the concentration of uric acid in the sample.

MCH and RDW

Complete Blood Count (CBC) with Five-Part Differential NHANES 2005–2006

The Beckman Coulter method of sizing and counting particles uses measurable changes in electrical

resistance produced by nonconductive particles suspended in an electrolyte.

A suspension of blood cells passes through a small orifice simultaneously with an electric current. A small opening (aperture) between electrodes is the sensing zone through which suspended particles pass. In the sensing zone, each particle displaces its volume of electrolyte. Beckman Coulter measures the displaced volume as a voltage pulse, the height of each pulse being proportional to the volume of the particle.

The quantity of suspension drawn through the aperture is for an exact reproducible volume. Beckman Coulter counts and sizes individual particles at a rate of several thousand per second. This method is independent of particle shape, color, and density.

The MAXM is a quantitative, automated, differential cell counter for in vitro diagnostic use.

The MAXM measures these parameters in whole blood: Cell	Parameter	Measured	Pulse size wavelength calculation	Reported units
WBC	white blood cell count	WBC bath	≥35 fL	*n* × 103 cells/µL
RBC	red blood cell count	RBC bath	36–360 fL	*n* × 106 cells/µL

Hgb	Hemoglobin concentration	WBC bath	525 nm	g/dL
Hct	hematocrit	computed	RBC x MCV/10	%
MCV	mean cell volume	derived from RBC histogram	# × size of RBC/total RBC	fL
MCH	mean cell hemoglobin	computer	Hgb/RBC × 10	pg
MCHC	mean cell hemoglobin concentration	computed	Hgb/Hct × 100	g/dL
RDW	red cell distribution width	derived from RBC histogram	CV expressed in % of the RBC size distribution	%
Plt	Platelet count	RBC bath	2 to 20 fL	*n* × 103 cells/μL
MPV	Mean platelet volume	derived from Plt histogram	Mean volume of Plt population under the fitted curve × constant	fL
NE%	neutrophil percent	derived from scatterplot	# cells inside NEarea/# cells inside total cell area × 100	%
NE #	neutrophil number	Absolute number	NE%/100 × WBC count	103 cells/μL

9. Beckman Coulter Reportable Range of Results

**Parameter**	**Linearity**	**Limits: The greater of**
WBC x 10^3 cells/ L	0 to 99.9	0.2 or 3.0%
RBC x 10^6 cells/ L	0 to 7.00	0.05 or 2.0%
Hgb g/dL	0 to 25.0	0.2 or 3.0%
MCV fL	50.0 to 150.0	2.0 or 3.0%
Plt x 10^3 cells/ L	0 to 999	10.0 or 7.0%
MPV fL	5.0 to 20.0	5.0%

Alkaline phosphatase

The DxC800 system uses a kinetic rate method using a 2-Amino-2-Methyl-1-Propanol (AMP) buffer to measure ALP activity in serum or plasma. In the reaction, the ALP catalyzes the hydrolysis of the colorless organic phosphate ester substrate, p-Nitrophenylphosphate, to the yellow colored product p-Nitrophenol and phosphate. This reaction occurs at an alkaline pH of 10.3. The system monitors the rate of change in absorbance at 410 nm over a fixed-time interval. This rate of change in absorbance is directly proportional to the ALP activity in the serum. (URL: https://wwwn.cdc.gov/Nchs/Nhanes/2011-2012/BIOPRO_G.htm)

Cotinine

Serum specimens and urine specimens are processed, stored, and shipped to the Division of Laboratory Sciences, National Center for Environmental Health, and Centers for Disease Control and Prevention for analysis.

Vials are stored under appropriate frozen (–20°C) conditions until they are shipped to National Center for Environmental Health for testing.

Cotinine is a major metabolite of nicotine that may be used as a marker for both active smoking, and as an index to Environmental Tobacco Smoke (ETS) exposure, or "passive smoking". Cotinine is generally preferred over nicotine for such assessments because of its substantially longer half-life. The half-life of cotinine in plasma has been estimated to be about 15–20 hrs; by contrast, the half-life of nicotine is only 0.5–3 hrs. Cotinine may be measured in serum, urine or saliva – the half-life of cotinine in all three fluids is essentially the same. Cotinine concentrations tend to be higher (3–8x) in urine than in serum; however, for studies requiring a quantitative assessment of exposure, plasma or serum is regarded as the fluid of choice. Therefore, serum was chosen for NHANES cotinine analyses.

Serum cotinine is measured by an isotope dilution-high performance liquid chromatography / atmospheric pressure chemical ionization tandem mass spectrometry (ID HPLC-APCI MS/MS). Briefly, the serum sample is spiked with methyl-D3 cotinine as an internal standard, and after an equilibration period, the sample is applied to a basified solid-phase extraction column. Cotinine is extracted off the column with methylene chloride, the organic extract is concentrated, and the residue is injected onto a short, C18 HPLC column. The eluant from these injections is monitored by APCI-MS/MS, and the m/z 80 daughter ion from the m/z 177 quasi-molecular ion is quantitated, along with additional ions for the internal standard, external standard, and for confirmation. Cotinine concentrations are derived from the ratio of native to labeled cotinine in the sample, by comparisons to a standard curve.

NNAL is measured by using liquid chromatography linked to tandem mass spectrometry (LC/MS/MS). For "total" NNAL assays, the urine sample is fortified with an NNAL-$^{13}C_6$ internal standard, and then hydrolyzed using β-glucuronidase in incubations for at least 24 hours. The samples are then extracted and cleaned up on a specially-designed solid-phase molecularly-imprinted polymer (MIP) column, after which the analyte is eluted and analyzed by LC/MS/MS, monitoring the m/z 210->180 native, and m/z 216->186 internal standard transition ions. NNAL concentrations are derived from the ratio of the integrated peaks of native to labeled ions by comparison to a standard calibration curve. Free NNAL measurements are conducted in a similar manner, but with the omission of prior enzymatic

hydrolysis. Bound NNAL (i.e. NNAL-Gluc) may be estimated from the difference of (Total NNAL – Free NNAL). This method has been described previously[24].

There were no changes (from the previous 2 years of NHANES) to equipment, lab methods, or lab site. Detailed instructions on specimen collection and processing can be found in the NHANES Laboratory/Medical Technologists Procedures Manual (LPM).

SUPPLEMENTAL METHODS 3: BIOCHEMICAL AND HEMATOLOGICAL INDICES SELECTED FOR HANDLS 2004-2018

Serum homocysteine

Serum homocysteine was measured on a sub-sample of HANDLS at visit 1 using a standard enzyme immunoassay conducted at the National Institute on Aging, Clinical Research Branch Core Laboratory, that is comparable to the method used for NHANES 1999 onwards.

Serum folate and vitamin B-12

Fasting blood was collected from each participant during MRV visits. Specimens in volumes of 2 mL serum were collected in small tubes and refrigerated. Serum folate and cobalamin were measured using chemiluminescence immunoassay by Quest Diagnostics, Chantilly, VA [25].

Serum creatinine

Using participant fasting venous blood specimens, baseline serum creatinine was measured at the National Institute on Aging, Clinical Research Branch Core Laboratory, using a modified kinetic Jaffe method (CREA method, Dade Dimension X-Pand Clinical Chemistry System, Siemens Healthcare Diagnostics Inc., Newark, DE) for a small group of participants (n=88); while the majority of participants (n=1,528) had baseline serum creatinine analyzed at Quest Diagnostics, Inc. by isotope dilution mass spectrometry (IDMS) (Olympus America Inc., Melville, NY) and standardized to the reference laboratory, Cleveland Clinic. While inter-assay coefficients of variation (CV) for this sample could not be calculated due to the use of only one or the other measurement of creatinine at baseline, only intra-assay CVs (mean/SD) could be estimated and those were 0.192 and 0.187 for the CREA and the IDMS methods, respectively. Serum creatinine was measured by Quest diagnostics in remaining waves.

RDW

RDW was measured by automated Coulter DXH 800 hematology analyzer as part of peripheral complete blood count (Beckman Coulter, Brea, CA), and was expressed as coefficient of variation (%) of red blood cell volume distribution. Regular calibration was performed every 3 months on the hematology analyzer and quality control was performed according to the manufacturer's recommendations.[26] There are usually two RDW measurements used for clinical purposes, namely the RDW-coefficient of variation (CV, unit: %), which we used in this study, and the RDW-Standard Deviation (SD, unit: fL) from which RDW-CV is derived. In fact, RDW-CV=RDW-SD×100/MCV, where MCV is the mean cell volume. The normal range for RDW-CV is 11.0 - 15.0%. Thus, the RDW-CV (%) depends on both the width of the distribution (normal range: 40-55 fL) curve and the MCV.[27]

MCH

The hematologic index MCH was calculated as follows: MCH = Hb/RBC. Using electronic cell sizing/cytometry/microscopy, Hb was assayed from a sample of 1 ml of blood drawn from participants after overnight fast and refrigerated up to 6 days (Quest diagnostics).

Serum uric acid (SUA)

SUA measurements are useful in the diagnosis and treatment of renal and metabolic disorders, including renal failure, gout, leukemia, psoriasis, starvation or other wasting conditions, and in patients receiving cytotoxic drugs. Using 1 ml of fasting blood serum, uric acid was measured using a standard spectrophotometry method. The reference range for adult men is 4.0-8.0 mg/dL, whereas for women, this range is cited as 2.5-7.0 mg/dL. (http://www.questdiagnostics.com/testcenter/TestDetail.action?ntc=905) Other reference ranges were also recently suggested and depend on the menopausal status of women. Those reference ranges are based on predictive value for gout outcomes among healthy individuals and do not necessarily predict other pathologies. Thus, based on recent research evidence, a "normal" SUA value is suggested to be <6.0 mg/dL for all healthy adult individuals.

Alkaline phosphatase

This liver enzyme was measured at Quest diagnostics using spectrophotometry. URL: https://testdirectory.questdiagnostics.com/test/test-detail/234/alkaline-phosphatase?cc=MASTER

SUPPLEMENTAL METHODS 4. DESCRIPTION OF TIME-INTERVAL MIXED-EFFECTS LINEAR REGRESSION MODELS, HANDLS 2004-2018

The main multiple mixed-effects regression models can be summarized as follows:

Multi-level models vs. **Composite models**

Eq. 1.1-1.4

$$Y_{ij} = \pi_{0i} + \pi_{1i} Time_{ij} + \varepsilon_{ij}$$

$$\pi_{0i} = \gamma_{00} + \gamma_{0a} X_{aij} + \sum_{k=1}^{l} \gamma_{0k} Z_{ik} + \zeta_{0i}$$

$$\pi_{1i} = \gamma_{10} + \gamma_{1a} X_{aij} + \sum_{m=1}^{n} \gamma_{1m} Z_{im} + \zeta_{1i}$$

$$Y_{ij} = \gamma_{00} + \gamma_{0a} X_{aij} + \sum_{k=1}^{l} \gamma_{0k} Z_{ik} + \gamma_{10} Time_{ij} + \gamma_{1a} X_{aij} Time_{ij} + \sum_{m=1}^{n} \gamma_{1m} Z_{im} Time_{ij} + (\zeta_{0i} + \zeta_{1i} Time_{ij} + \varepsilon_{ij})$$

Where Y_{ij} is the outcome (Each biochemical or hematological marker measured at visits 1, 2 and 3) for each individual "i" and visit "j"; π_{0i} is the level-1 intercept for individual i; π_{1i} is the level-1 slope for individual i; γ_{00} is the level-2 intercept of the random intercept π_{0i} ; γ_{10} is the level-2 intercept of the slope π_{1i} ; Z_{ik} is a vector of fixed covariates for each individual *i* that are used to predict level-1 intercepts and slopes and included baseline age (Age_{base}) among other covariates. X_{ija}, represents the main predictor variable (Serum Hcy at visit 1); ζ_{0i} and ζ_{1i} are level-2 disturbances; ε_{ij} is the within-person level-1 disturbance. Of primary interest are the main effects of each exposure X_a (γ_{0a}) and their interaction with *TIME* (γ_{1a}), as described in a previous methodolgical paper.[28]

Supplementary Table 1. Predictive models of serum homocysteine (Log$_e$ transformed): CV, minimum BIC and adaptive linear LASSO for NHANES III, phase 2: training half-sample results and consistency with testing half-sample, 30-65 y.

	cvLASSO	Min BIC LASSO	Adaptive LASSO	Between sample R^2: cvLASSO	Between sample R^2: Min BIC	Between sample R^2: Adaptive LASSO
**λ**						
**Training Sample, N=2004**	0.0067622	0.047706	0.4496467	0.47	0.51	0.47
**Testing Sample, N=2004**	--	--	--	0.47	0.49	0.48
**Non-zero parameter predictors (Log$_e$ transformed, z-scored, SI units), training set (descending order of effect size)**						
**Serum folate**	x	x	X[a]			
**Serum creatinine**	x	x	X[a]			
**Age, y**	x	x	X[a]			
**Serum vitamin B-12**	x	x	X[a]			
**Aspartate aminotransferase**	x	x	x			
**Alanine aminotransferase**	x		X[a]			
**Serum uric acid**	x	x	X[a]			
**Mean cell hemoglobin**	x		X[a]			

**Serum albumin**	**x**	**x**	**X[a]**
**Census region: South vs. NorthEast**	**x**	**x**	**X[a]**
**Serum total calcium**	**x**	**x**	**X[a]**
**Lead**	**x**	**x**	**x**
**Serum triglycerides**	**x**		**x**
**Platelet count**	**x**		**X[a]**
**Gamma glutamyl transferase**	**x**		**x**
**Serum iron**	**x**		**x**
**Serum sodium**	**x**		**X[a]**
**Race/ethnicity: Other vs. NH white**	**x**	**x**	**X[a]**
**RBC folate**	**x**	**x**	**x**
**Serum vitamin A**	**x**		**x**
**Serum glucose**	**x**	**x**	**X[a]**
**Serum potassium**	**x**		**x**
**Serum total bilirubin**	**x**		**x**
**Serum vitamin C**	**x**		**x**
**White blood cell count**	**x**		**X[a]**
**Serum total calcium**	**x**	**x**	**x**
**Serum phosphorus**	**x**		**X[a]**
**Urinary albumin**	**x**		**X[a]**

**Serum selenium**	x		x
**Red cell distribution width**	x		x
**Serum normalized calcium**	x	x	x
**Below vs. Above poverty**	x	x	X[a]
**Serum alkaline phosphatase**	x	x	X[a]
**Serum thyroxine**	x		x
**Serum lactate dehydrogenase**	x		X[a]
**Serum sum retinyl esters**	x	x	X[a]
**Serum cotinine, ng/mL**	x	x	X[a]
**Serum bicarbonate**	x		X[a]
**Urinary iodine**	x		x
**Urinary creatinine**	x		x
**Rural vs. Urban**	x	x	X[a]
**Sex**	x	x	X[a]
**Serum vitamin D**	x		x
**Glycated hemoglobin, %**	x		
**Serum cholesterol**	x		
**Serum hepatitis C antibody**	x		
**Mean cell volume**	x	x	
**West vs. NorthEast**	x	x	X[a]
**Serum globulin**	x		

**Serum thyroid stim hormone**	x		
**Serum lutein/zeaxanthin**	x		
**Mexican-American vs. NH whites**	x	x	X[a]
**Serum HDL cholesterol**	x		[a]
**Serum chloride**	x		[a]
**Serum rubella antibody**	x		
**Serum anti-thyroglobulin antibody**	x		
**Serum hepatitis A antibody**	x		
**Platelet distribution width**	x		
**Vitamin A**			[a]
**Serum antimicrosomal antibody**			[a]
**RBC count**			[a]
**Intercept**	x	x	x

Abbreviations: BIC=Bayesian Information Criterion; cv=cross-validation; Hcy=Homocysteine; LASSO= least absolute shrinkage and selection operator ; NH=Non-Hispanic; NHANES=National Health and Nutrition Examination Surveys.

[a] Non-zero terms identified in the same half sample using adaptive LASSO logit for binary Hcy outcome.

Supplementary Table 2. Reduced selected LASSO model for biochemical and hematological predictors (Log_e transformed and z-scored) of Hcy (Log_e transformed and z-scored) and elevated Hcy (equivalent to Hcy>14 *µmol/L*), adjusting for socio-demographic factors: NHANES III, phase 2; ages 30-65y [1]

	**Homocysteine, Log_e transformed, z-score**			**Elevated homocysteine: >14 µmol/L vs. ≤14 µmol/L**		
	β	(SE)	P-value	OR	95% CI	P-value
**Reduced model**	(N=3,784)			(N=3,794)		
Serum folate	**-0.36**	**(0.04)**	**<0.001**	**0.29**	**(0.18,0.45)**	**<0.001**
Serum creatinine	**+0.26**	**(0.03)**	**<0.001**	**1.85**	**(1.30,2.65)**	**0.002**
Age	**+0.19**	**(0.03)**	**<0.001**	**1.26**	**(1.02,1.57)**	**0.037**
Serum vitamin B-12	**-0.23**	**(0.06)**	**0.001**	**0.50**	**(0.31,0.83)**	**0.009**
Aspartate aminotransferase	**+0.11**	**(0.02)**	**<0.001**	**1.80**	**(1.25;2.59)**	**0.003**
Alanine aminotransferase	**-0.16**	**(0.02)**	**<0.001**	**0.39**	**(0.27,0.57)**	**<0.001**
Serum uric acid	**+0.11**	**(0.02)**	**<0.001**	**1.48**	**(1.10,2.00)**	**0.013**
Mean cell hemoglobin	**+0.11**	**(0.03)**	**0.004**	**1.69**	**(1.22,2.34)**	**0.003**
Serum albumin	**+0.17**	**(0.02)**	**<0.001**	**1.77**	**(1.33,2.36)**	**<0.001**
Total calcium	**+0.06**	**(0.02)**	**0.006**	**…** [2]		
Platelet count	**-0.04**	**(0.02)**	**0.096**	**…** [2]		
Gamma glutamyl transferase	**+0.04**	**(0.02)**	**0.081**	**…** [2]		
Serum glucose	**-0.05**	**(0.02)**	**0.009**	**…** [2]		
Serum vitamin C	**+0.05**	**(0.02)**	**0.058**	**…** [2]		
Red cell distribution width	**+0.11**	**(0.02)**	**<0.001**	**1.32**	**(1.09,1.61)**	**0.008**
Alkaline phosphatase	**+0.08**	**(0.03)**	**0.013**	**2.29**	**(1.52,3.44)**	**<0.001**
Serum retinyl esters	**-0.05**	**(0.02)**	**0.054**	**0.79**	**(0.65,0.96)**	**0.009**
Serum cotinine	**+0.07**	**(0.02)**	**0.012**	**1.27**	**(1.07,1.50)**	**<0.001**

Abbreviations: Hcy=Homocysteine; LASSO= least absolute shrinkage and selection operator ; NH=Non-Hispanic; NHANES=National Health and Nutrition Examination Surveys; RBC=Red blood cells.

[1] Reduced models were determined using backward elimination starting from the initially selected LASSO model, applied to both linear and logistic regression models. At each step of the backward elimination, only parameters with p<0.10 were retained. The final step include parameters with p<0.10. [2] Predictor eliminated as compared to the linear regression reduced model. See units in Table 1.

Supplementary Figure 2. ROC curves of selected correlates of elevated homocysteine (>14 *μmol/L*) and cut-point determination: NHANES III, phase 2; NHANES 1999-2006 and pooled NHANES

(A) Lower Serum Folate

NHANES III, phase 2[1] **NHANES 1999-2006[2]** **Pooled NHANES[3]**

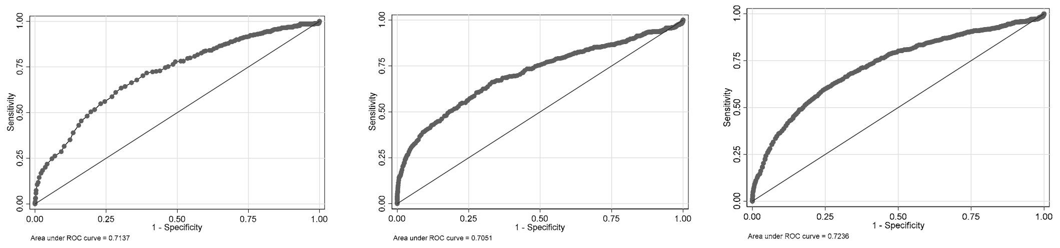

[1] Optimal cut-point at -2.20; [2] Optimal cut-point at -3.07; [3] Optimal cut-point at -2.83.

Note: Serum folate is in SI units (*nmol/L*), was Log_e transformed and multiplied by -1. Thus a value ≥-2.83 on this scale, corresponds to a Log_e transformed value ≤+2.83 and an untransformed value ≤16.93 *nmol/L*.

(B) Higher Serum Creatinine

NHANES III, phase 2[1] **NHANES 1999-2006[2]** **Pooled NHANES[3]**

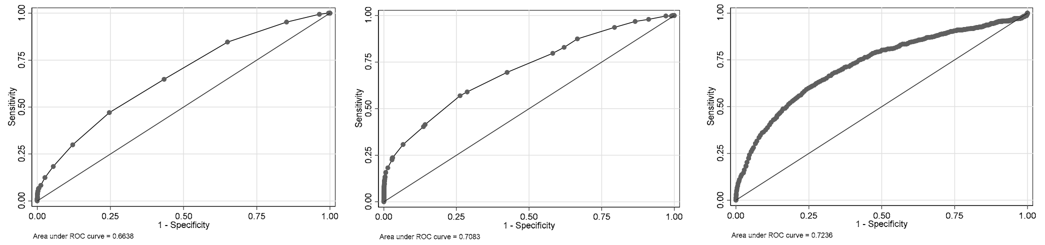

[1] Optimal cut-point at +4.576; [2] Optimal cut-point at +4.376; [3] Optimal cut-point at +4.481.

Note: Serum creatinine is Log_e transformed. Thus, a value ≥4.481 corresponds to an untransformed value ≥88.3. The same applies to all other biomarkers, except for age which not transformed. See units in Table 1.

(C) Older Age

NHANES III, phase 2[1] **NHANES 1999-2006**[2] **Pooled NHANES**[3]

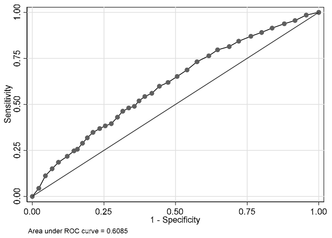

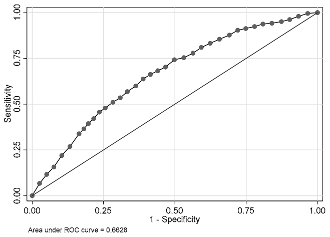

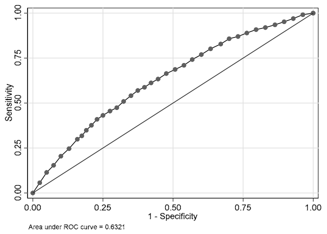

[1] Optimal cut-point at 46 years; [2] Optimal cut-point at 50; [3] Optimal cut-point at 49.

(D) Lower Serum B-12

NHANES III, phase 2[1] **NHANES 1999-2006**[2] **Pooled NHANES**[3]

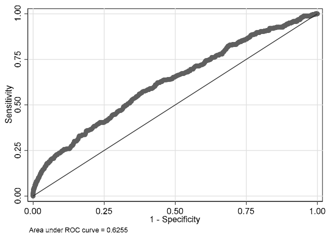

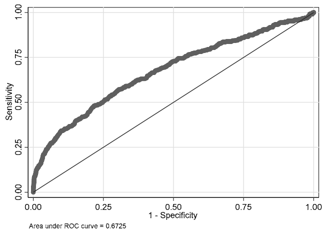

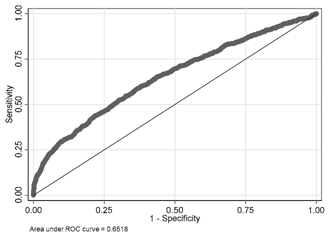

[1] Optimal cut-point at -5.76; [2] Optimal cut-point at -5.72; [3] Optimal cut-point at -5.74.

Note: Serum vitamin B-12 is in SI units (*pmol/L*), was Log_e transformed and multiplied by -1. Thus a value ≥-5.74 on this scale, corresponds to a Log_e transformed value ≤+5.74 and an untransformed value ≤311 *pmol/L.*

(E) Higher MCH

NHANES III, phase 2[1] **NHANES 1999-2006**[2] **Pooled NHANES**[3]

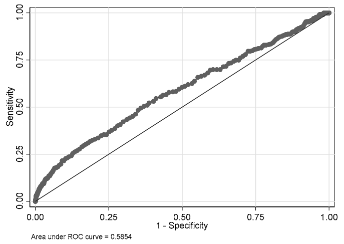

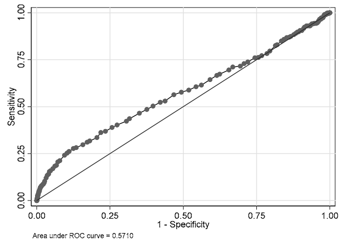

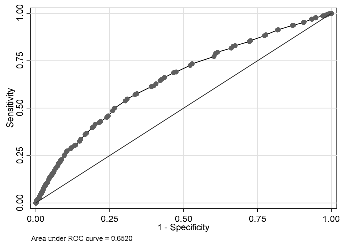

[1] Optimal cut-point at +3.409; [2] Optimal cut-point at +3.430; [3] Optimal cut-point at +3.422.

Note: MCH is in SI units (pg) and was Log_e transformed.

(F) Higher RDW

NHANES III, phase 2[1] **NHANES 1999-2006[2]** **Pooled NHANES[3]**

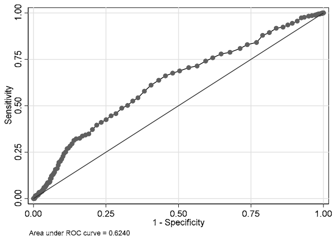

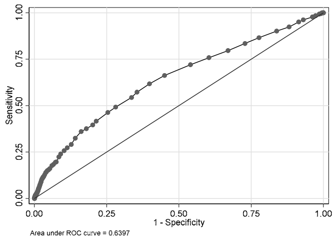

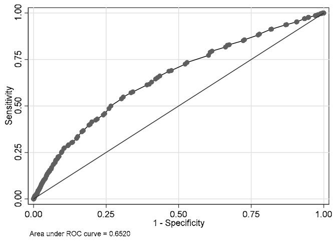

[1] Optimal cut-point at +2.576; [2] Optimal cut-point at +2.541; [3] Optimal cut-point at +2.553.

Note: RDW is in SI units (%) and was Log_e transformed.

(G) Higher Serum Uric acid

NHANES III, phase 2[1] **NHANES 1999-2006[2]** **Pooled NHANES[3]**

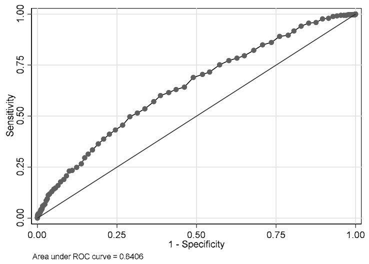

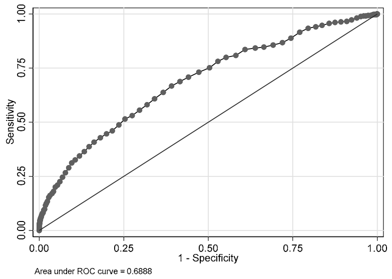

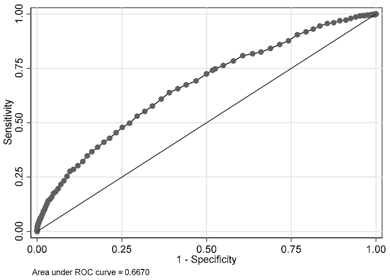

[1] Optimal cut-point at 5.808; [2] Optimal cut-point at 5.826; [3] Optimal cut-point at 5.826.

Note: Serum uric acid is in SI units ($\mu mol/L$) and was Log_e transformed.

(H) Higher Alkaline phosphatase

NHANES III, phase 2[1] **NHANES 1999-2006[2]** **Pooled NHANES[3]**

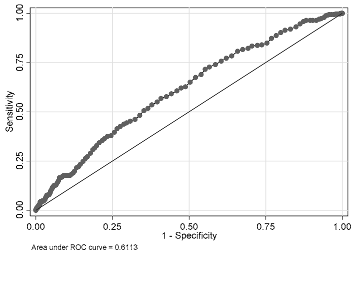

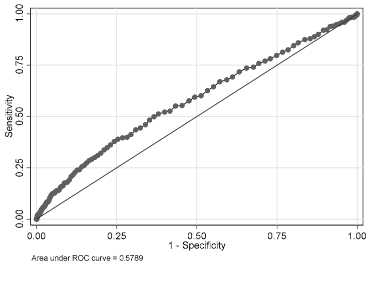

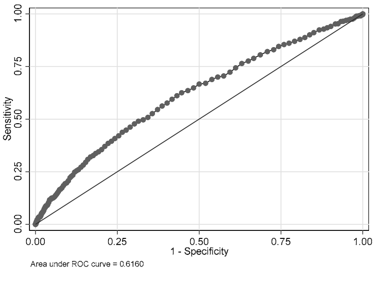

[1] Optimal cut-point at 4.477; [2] Optimal cut-point at 4.290; [3] Optimal cut-point at 4.356.

Note: Alkaline phosphatase is in SI units (*U/L*) and was Log_e transformed.

(I) Higher serum Cotinine

NHANES III, phase 2[1] **NHANES 1999-2006[2]** **Pooled NHANES[3]**

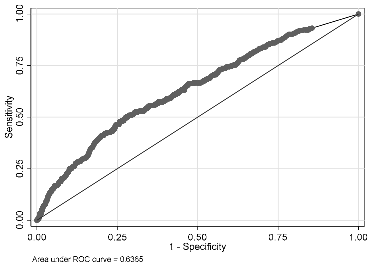

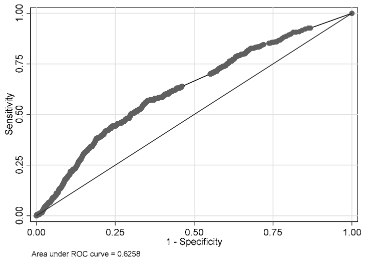

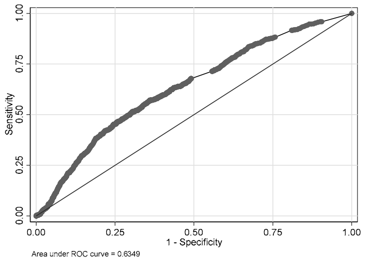

[1] Optimal cut-point at -0.349; [2] Optimal cut-point at -0.778; [3] Optimal cut-point at -0.579.

Note: Serum cotinine is in SI units *(ng/mL)* and was Log_e transformed.

Supplementary references

1. Jacques, P.F.; Rosenberg, I.H.; Rogers, G.; Selhub, J.; Bowman, B.A.; Gunter, E.W.; Wright, J.D.; Johnson, C.L. Serum total homocysteine concentrations in adolescent and adult Americans: results from the third National Health and Nutrition Examination Survey. *Am J Clin Nutr* **1999**, *69*, 482-489, doi:10.1093/ajcn/69.3.482.
2. Araki, A.; Sako, Y. Determination of free and total homocysteine in human plasma by high-performance liquid chromatography with fluorescence detection. *J Chromatogr* **1987**, *422*, 43-52, doi:10.1016/0378-4347(87)80438-3.
3. (DHHS)., U.S.D.o.H.a.H.S. National Center for Health Statistics. NHANES III reference manuals and reports. Hyattsville, MD: Centers for Disease Control and Prevention, 1996. . **1996**.
4. Gunter, E.W., Lewis, B. G., Koncikowski, S. M., . *Laboratory Procedures Used for the Third National Health and Nutrition Examination Survey (NHANES III), 1988-1994*, http://www.cdc.gov/nchs/data/nhanes/nhanes3/cdrom/nchs/manuals/labman.pdf; US DHHS, Public Health Service, Centers for Disease Control and Prevention, National Center for Environmental Health and National Center for Health Statistics: Atlanta, GA and Hyattsville, MD, 2010.
5. Addison, G.M.; Beamish, M.R.; Hales, C.N.; Hodgkins, M.; Jacobs, A.; Llewellin, P. An immunoradiometric assay for ferritin in the serum of normal subjects and patients with iron deficiency and iron overload. *Journal of clinical pathology* **1972**, *25*, 326-329.

6. Laboratories, B.-R. *Instruction Manual, Bio-Rad Quantaphase Folate Radioassay Kit. ;* Hercules, CA, 1987.
7. 7+7, N. *Electrolyte Analyzer instruction manual.*; Waltham (MA), 1986.
8. Lewis, S.A.; Hardison, N.W.; Veillon, C. Comparison of isotope dilution mass spectrometry and graphite furnace atomic absorption spectrometry with Zeeman background correction for determination of plasma selenium. *Analytical chemistry* **1986**, *58*, 1272-1273, doi:10.1021/ac00297a070.
9. Paschal D. C., K.M.M. Automated direct determination of selenium in serum by electrothermal atomic absorption spectroscopy. *At Spectrosc* **1986**, *7*, 75-78.
10. Plan and operation of the Third National Health and Nutrition Examination Survey, 1988-94. Series 1: programs and collection procedures. *Vital and health statistics. Ser. 1, Programs and collection procedures* **1994**, 1-407.
11. World Health Organization. Diabetes Mellitus: Report of a WHO study group. In *WHO Technical Report Series* Geneva, Switzerland, 1995; p 727.
12. Bull, B.S.; Rittenbach, J.D. A proposed reference haematocrit derived from multiple MCHC determinations via haemoglobin measurements. *Clinical and laboratory haematology* **1990**, *12 Suppl 1*, 43-53.
13. National Committee for Clinical Laboratory Standards, N.d.H.-W., PA: NCCLS. ; 1993. *Procedure for determining packed cell volume by the microhematocrit method -- second edition: approved standard.*; NCCLS: Wayne, PA, 1993.
14. Benotti, J.; Benotti, N. Protein-Bound Iodine, Total Iodine, and Butanol-Extractable Iodine by Partial Automation. *Clinical chemistry* **1963**, *12*, 408-416.
15. Benotti, J.; Benotti, N.; Pino, S.; Gardyna, H. Determination of total iodine in urine, stool, diets, and tissue. *Clinical chemistry* **1965**, *11*, 932-936.
16. Chavers, B.M.; Simonson, J.; Michael, A.F. A solid phase fluorescent immunoassay for the measurement of human urinary albumin. *Kidney international* **1984**, *25*, 576-578.
17. Pruszkowska, E.; Carnrick, G.R.; Slavin, W. Direct determination of cadmium in urine with use of a stabilized temperature platform furnace and Zeeman background correction. *Clinical chemistry* **1983**, *29*, 477-480.
18. Center for Disease Control and Prevention (CDC). National Health and Nutrition Examination Survey. Availabe online: http://www.cdc.gov/nchs/nhanes.htm. (accessed on October 25).
19. Shipchandler, M.T.; Moore, E.G. Rapid, fully automated measurement of plasma homocyst(e)ine with the Abbott IMx analyzer. *Clinical chemistry* **1995**, *41*, 991-994.
20. Boushey, C.J.; Beresford, S.A.; Omenn, G.S.; Motulsky, A.G. A quantitative assessment of plasma homocysteine as a risk factor for vascular disease. Probable benefits of increasing folic acid intakes. *Jama* **1995**, *274*, 1049-1057, doi:10.1001/jama.1995.03530130055028.
21. Ueland, P.M.; Refsum, H.; Stabler, S.P.; Malinow, M.R.; Andersson, A.; Allen, R.H. Total homocysteine in plasma or serum: methods and clinical applications. *Clinical chemistry* **1993**, *39*, 1764-1779.
22. (CDC), C.f.D.C.a.P. Centers for Disease Control and Prevention (CDC). National Health and Nutrition Examination Surveys (NHANES 2005-06): Description of Laboratory Methodology: Vitamin B-12. Availabe online:

http://www.cdc.gov/nchs/nhanes/nhanes2005-2006/B12_D.htm#Description_of_Laboratory_Methodology (accessed on February 15).
23. (CDC), C.f.D.C.a.P. National Health and Nutrition Examination Surveys (NHANES 2005-06): Description of Laboratory Methodology: Folate. Availabe online: ttp://www.cdc.gov/nchs/nhanes/nhanes2005-2006/FOLATE_D.htm#Description_of_Laboratory_Methodology (accessed on February 15).
24. Xia, Y.; McGuffey, J.E.; Bhattacharyya, S.; Sellergren, B.; Yilmaz, E.; Wang, L.; Bernert, J.T. Analysis of the tobacco-specific nitrosamine 4-(methylnitrosamino)-1-(3-pyridyl)-1-butanol in urine by extraction on a molecularly imprinted polymer column and liquid chromatography/atmospheric pressure ionization tandem mass spectrometry. *Analytical chemistry* **2005**, *77*, 7639-7645, doi:10.1021/ac058027u.
25. Diagnostics, Q. Vitamin B-12 (cobalamin) and folate panel. Availabe online: https://testdirectory.questdiagnostics.com/test/test-detail/7065/vitamin-b12-cobalamin-and-folate-panel-serum?cc=MASTER (accessed on October 21st).
26. Diagnostics, Q. Hemogram. Availabe online: https://www.questdiagnostics.com/testcenter/BUOrderInfo.action?tc=7008&labCode=DAL (accessed on May 13sth).
27. techs, O.l.c.e.f.c.l.a.m. Red Blood Cell Distribution Width (RDW): Definition and Calculation. **2019**.
28. Blackwell, E.; de Leon, C.F.; Miller, G.E. Applying mixed regression models to the analysis of repeated-measures data in psychosomatic medicine. *Psychosom Med* **2006**, *68*, 870-878, doi:01.psy.0000239144.91689.ca [pii]

10.1097/01.psy.0000239144.91689.ca.
